# Bacterial mutation dynamics emerging insights into virulence evolution and drug resistance. A review study

**DOI:** 10.3389/fmed.2026.1863965

**Published:** 2026-06-17

**Authors:** Sulaiman A. Alsalamah

**Affiliations:** Department of Biology, College of Science, Imam Mohammad Ibn Saud Islamic University (IMSIU), Riyadh, Saudi Arabia

**Keywords:** antimicrobial resistance, bacterial mutations, mutation, virulence, virulence determinants

## Abstract

**Introduction:**

Bacterial mutations are a fundamental driver of microbial evolution, enabling rapid adaptation to environmental stress and antimicrobial exposure. Genetic alterations may arise spontaneously or be induced by physical, chemical, or biological factors, generating phenotypic diversity that influences virulence, pathogenicity, and antimicrobial resistance. Understanding mutation dynamics is essential for predicting bacterial adaptation and addressing the growing threat of antimicrobial resistance.

**Methods:**

This review synthesizes current knowledge on the molecular mechanisms, evolutionary patterns, and biological consequences of bacterial mutations. Relevant literature was examined to evaluate the roles of spontaneous and induced mutations, mutational biases, virulence evolution, and antimicrobial resistance development. Emerging genomic technologies used to investigate mutation dynamics were also assessed.

**Results and Discussion:**

Evidence indicates that bacterial mutations contribute significantly to adaptive evolution by generating genetic variation upon which natural selection acts. Although mutations are undirected with respect to fitness, their distribution across genomes is influenced by mutational biases and DNA repair mechanisms. Mutation-driven genetic changes were found to play a critical role in enhancing virulence traits and promoting antimicrobial resistance. Furthermore, advanced genomic approaches, including CRISPR-based genome editing and single-cell sequencing, have expanded the ability to characterize mutation processes at high resolution. The findings highlight the importance of understanding both the mechanisms and biases of bacterial mutations in shaping evolutionary trajectories. The interplay between mutation-driven virulence evolution and antimicrobial resistance underscores the need for continued surveillance and molecular investigation. Emerging genomic technologies offer promising opportunities to predict bacterial adaptation and develop innovative strategies for controlling drug-resistant pathogens.

## 1 Introduction

Bacterial genomes typically consist of a single double-stranded circular DNA molecule, ranging from approximately 0.5 to 10 Mb (with an average around 4 Mb in well-studied species like *Escherichia coli*), and are organized into operons—clusters of genes transcribed as a single mRNA. Genome size and organization vary substantially across

bacterial species. A genetic mutation is a change in the nucleotide sequence of DNA that can either impair normal cellular functions or confer new traits, such as antibiotic resistance or altered virulence. The seminal work of Luria and Delbrück (1) using the fluctuation test established that mutations arise spontaneously and randomly in bacterial populations, occurring independently of selective pressure—a finding that revolutionized our understanding of bacterial adaptation. Mutations can arise spontaneously due to errors during DNA replication or can be induced by chemical, physical, or biological mutagens (1, 2). Bacterial cells can acquire new phenotypes through horizontal gene transfer, and each bacterial genome contains a vast array of genes that confer resilience against changing environmental conditions. However, mutation frequently produces unique adaptive phenotypes when populations are subjected to novel selection pressures. (3). The term “wild type” refers to organisms chosen as reference strains, whereas “mutants” refers to their offspring that have undergone changes. Differential media separate wild type and mutant strains according to other phenotypic characteristics, while selective media separate them according to growth (4).

The capacity for adaptation of bacteria through mutation is extensively established and well recognized (5) and it has led to antibiotic resistance which is such a serious danger (6). As a result, a lot of academics are committed to clarifying the effects of mutation, including how the networks of gene regulation are rearranged and how protein product functions change after a mutation. The scientific investigation of the mutation processes itself is a complementing endeavor to this process. Because we will be capable to predict the occurrence of mutations if we can comprehend how they occur. This will enable us to make precise short-term evolutionary predictions, putting us in a proactive position to address the effects of mutation immediately as they arise (7).

One of the sources of genetic variations is mutation, which is frequently referred to as a stochastic force. This indicates that the mutation mechanism depends on random occurrences. But chance does not imply that every mutation has an equal chance of occurring. Mutations are biased instead. They happen at varying rates, with some mutation types occurring more frequently than others (8) and some genomic regions (e.g., those further away from the point of replication being more malleable than others. Because of this, the genetic variation-generating and adaptation-facilitating raw material is distributed unevenly, causing some mutations to occur in the entire population more frequently than others. Even though they are frequently disregarded, these consequences can significantly affect how adaptation and evolution proceed. Bacterial adaptability and mutation bias can occasionally have an adversarial relationship because the kinds of mutations that mutation bias promotes may be directly at odds with those that are selective advantageous. Despite the fact that bacterial genomes are usually biased toward mutations from GC to AT, some bacterial genomes are still GC-rich (9).

Mutation bias and natural selection can work together to increase the likelihood of some outcomes over others when mutation frequencies are greater at a nucleotide site that can provide an adaptive benefit. Localized mutational “hotspots” inside bacterial genes create adaptive consequences, demonstrating how local and particular these mutational tendencies can happen (10). According to recent research by Cano et al. (11) adaptive consequences for *Mycobacterium tuberculosis* and *Escherichia coli* follow known biased mutational. Recent studies have shown that mutation bias affects adaptability in the bacterial sector (12), which gives microbiologists from different fields a chance to think about how mutation bias affects the development of their bacteria or bacteria of relevance. This review addresses the following question: How do the mechanisms, rates, and biases of bacterial mutations influence the evolution of virulence and antimicrobial resistance, and can this knowledge be translated into predictive or therapeutic strategies? While the review covers fundamental mutation mechanisms in bacteria, the primary focus is on clinically relevant outcomes—specifically, how mutation dynamics enable pathogens to adapt to host defenses and antibiotic pressure. The central argument is that mutation bias and environmental stress interplay to produce predictable evolutionary trajectories, and that emerging genomic technologies can exploit this predictability to combat resistance. Readers should take away that mutations are not simply stochastic noise; they are a structured, often biased, and potentially foreseeable driver of bacterial pathogen evolution. This review will critically examine the mechanisms of bacterial mutations, evaluate evolutionary implications and clinical relevance of mutation dynamics, synthesize current knowledge of virulence gene mutations and their consequences, analyze potential interactions between mutant and non-mutant strains, and discuss emerging technologies for studying and potentially directing bacterial mutation outcomes.

## 2 Methods

### 2.1 Search strategy

A comprehensive literature search was conducted to identify relevant studies on bacterial mutation dynamics, virulence evolution, and antimicrobial resistance. The following electronic databases were systematically searched: PubMed/MEDLINE, Scopus, Embase, and Web of Science. The search covered the period from January 1990 to March 2025 to capture both foundational historical studies and recent advances. Seminal papers before 1990 were identified through citation tracking and historical literature reviews. This review focuses on **pathogenic bacteria** and emphasizes **clinically relevant mutations**—those affecting drug resistance and virulence. However, the underlying mutation mechanisms (spontaneous, induced, biased) are general to all bacteria, and examples from non-pathogenic model organisms (e.g., Escherichia coli laboratory strains) are included where they illuminate fundamental principles.

The search strategy employed combinations of the following keywords using Boolean operators: (“bacterial mutation” OR “spontaneous mutation” OR “induced mutation”) AND (“virulence” OR “pathogenicity” OR “virulence factors”) AND (“antimicrobial resistance” OR “antibiotic resistance” OR “drug resistance”) AND (“evolution” OR “adaptation” OR “fitness”).

Additional searches were conducted in specialized databases: Mutation Spectra Database (http://info.med.yale.edu/mutbase/), Comprehensive Antibiotic Resistance Database (CARD) (https://card.mcmaster.ca/), and Microbial Genome Database (MBGD) (https://mbgd.nibb.ac.jp/) for comparative genomic analysis (13).

### 2.2 Inclusion and exclusion criteria

Studies were included if they met the following criteria: Original research articles, systematic reviews, or meta-analyses published in peer-reviewed journals, English language publications, Studies investigating bacterial mutations at the molecular, cellular, or population level, Research examining relationships between mutations and virulence or resistance phenotypes, and *in vitro, in vivo*, or *in silico* studies with clearly described methodology.

Studies were excluded if they: Were conference abstracts, case reports, editorials, or opinion pieces without original data, focused on non-bacterial organisms (viruses, fungi, parasites), examined mutations in non-microbial contexts (cancer genetics, eukaryotic genetics), and lacked clear experimental methodology or insufficient data for interpretation.

### 2.3 Screening and selection process

The literature screening followed PRISMA (Preferred Reporting Items for Systematic Reviews and Meta-Analyses) guidelines (115). The selection process involved:

(1) Title and abstract screening: Two independent reviewers screened all titles and abstracts against inclusion criteria. Disagreements were resolved through discussion or consultation with a third reviewer. (2) Full-text assessment: Potentially relevant articles were retrieved in full text and assessed for eligibility using a standardized form. (3) Reference list checking: Reference lists of included articles and relevant reviews were manually searched for additional eligible studies.

**Figure 1:**
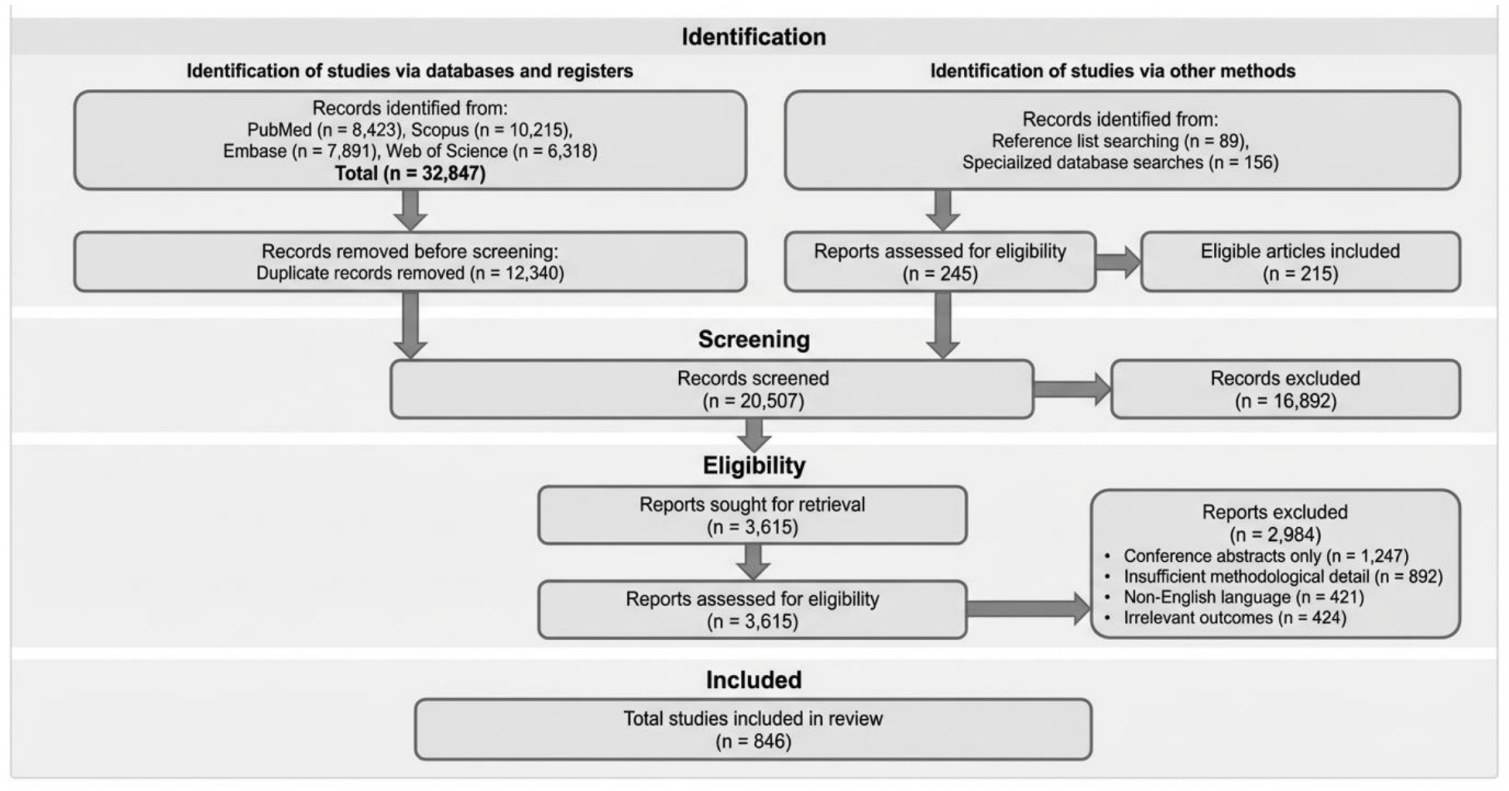
PRISMA flow diagram illustrating the literature selection process for this review.

The initial database search yielded 32,847 records: PubMed (*n* = 8,423), Scopus (*n* = 10,215), Embase (*n* = 7,891), and Web of Science (*n* = 6,318). After removing duplicates (*n* = 12,340), 20,507 records underwent title and abstract screening. Title and abstract screening excluded 16,892 records that clearly did not meet inclusion criteria (non-bacterial studies, non-mutation research, irrelevant outcomes). Full-text articles (*n* = 3,615) were assessed for eligibility. Of these, 2,984 were excluded for the following reasons: conference abstracts only (*n* = 1,247), insufficient methodological detail (*n* = 892), non-English language (*n* = 421), irrelevant outcomes (*n* = 424). Additional records identified through reference list searching (*n* = 89) and specialized database searches (*n* = 156) were screened and assessed, yielding 215 additional eligible articles. A total of 846 studies met all inclusion criteria and were included in this narrative review (Figure 1). The included studies were categorized into four thematic groups: (i) mutation mechanisms, (ii) virulence-associated mutations, (iii) antibiotic resistance mutations and (iv) emerging mutation detection technologies.

### 2.4 Data extraction and synthesis

Data were extracted using a standardized data extraction form that captured: Study characteristics (author, year, country, study design), Bacterial species and strains investigated, mutation types and mechanisms, virulence factors and associated genes, resistance mechanisms and associated genes, and key findings and conclusions.

### 2.5 Justification for narrative synthesis approach

A quantitative meta-analysis was not appropriate for this review due to several factors:

(1) Heterogeneity of study designs: Included studies employed diverse methodologies including *in vitro* evolution experiments, clinical surveillance studies, molecular characterization, and computational predictions, making statistical pooling invalid. (2) Diversity of bacterial species: Studies covered numerous bacterial pathogens with distinct genetic backgrounds, mutation rates, and evolutionary trajectories, precluding meaningful quantitative comparison. (3) Variability in outcome measures: Outcomes were reported using different metrics (mutation frequencies, resistance levels, fitness costs, virulence scores) that cannot be directly standardized. (4) Temporal evolution of the field: The literature spans 35 years, during which detection methods, definitions, and reporting standards have substantially changed. Therefore, a thematic narrative synthesis was selected as the most appropriate approach to integrate findings across diverse study types while maintaining scientific rigor. This approach allows for critical evaluation of evidence quality, identification of knowledge gaps, and synthesis of overarching principles that emerge from the collective literature.

### 2.6 Quality assessment

The quality of included studies was assessed using appropriate tools: SYRCLE's Risk of Bias tool for animal studies, Newcastle-Ottawa Scale for observational studies, and SIGN checklists for experimental studies. Studies were not excluded based on quality scores, but quality considerations were incorporated into the narrative synthesis and interpretation of findings.

### 2.7 Figure preparation

Figures were prepared using graphical tools and assisted by SciSpace for layout optimization and formatting to ensure optimal visual (https://scispace.com/) clarity and accurate representation of complex molecular mechanisms. The scientific content and interpretation were developed and verified by the authors.

### 2.8 Current standards and emerging technologies for mutagenesis prediction and detection

Recent advances in high-throughput experimental techniques and computational analytics have transformed bacterial mutagenesis from a descriptive discipline to a predictive one. The ability to forecast mutational outcomes, identify resistance-conferring variants before clinical emergence, and manipulate mutation dynamics is increasingly within reach. Several core technologies constitute the current standard for mutagenesis detection and prediction (14).

#### 2.8.1 Next-generation sequencing (NGS): short-read and long-read approaches

NGS has become the cornerstone of modern bacterial mutation detection (13). Short-read sequencing (Illumina platforms) provides high per-base accuracy (>99.9%) and deep coverage, making it optimal for single nucleotide variant (SNV) calling and quantification of rare variants within heterogeneous populations. Long-read sequencing (Oxford Nanopore, PacBio) spans repetitive regions, resolves structural variants, and facilitates complete genome assembly. Hybrid approaches combining short and long reads are increasingly recommended for comprehensive mutation detection (15). Targeted NGS (tNGS) is now positioned as a key tool for drug resistance profiling in *Mycobacterium tuberculosis* (16).

#### 2.8.2 Deep mutational scanning (DMS)

Deep mutational scanning combines saturation mutagenesis, functional selection, and high-throughput sequencing to generate comprehensive fitness landscapes—mapping how all possible mutations in a gene affect function. In bacteriology, DMS has been applied to map resistance-associated mutations in antibiotic targets and predict clinical variant outcomes before they emerge (7). Integration of DMS with CRISPR-based mutagenesis enables precise tracking of mutational trajectories (17). Standardized analysis protocols are essential for reproducibility across experiments (18).

#### 2.8.3 MALDI-TOF proteomics for resistance profiling

Matrix-Assisted Laser Desorption/Ionization Time-of-Flight Mass Spectrometry (MALDI-TOF MS) offers rapid, cost-effective complement to genomics for indirect detection of resistance-associated mutations via protein expression signatures. Distinct MALDI-TOF MS spectral profiles differentiate methicillin-resistant *Staphylococcus aureus* (MRSA) from susceptible strains using biomarkers such as PSM-mec (2,414 Da) and delta toxin (3,006 Da) (19). When integrated with machine learning algorithms, spectral data can predict resistance phenotypes in under 1 h (20). The technology's low cost and established presence in clinical laboratories make it attractive for screening known resistance mechanisms (21).

#### 2.8.4 Bioinformatics tools and genomic pipelines

Bioinformatics pipelines are essential for analyzing NGS data. Standard workflows include read preprocessing (FastQC), genome assembly (SPAdes), annotation (Prokka), and resistance gene identification using databases such as CARD and ResFinder (22). Deep learning tools such as SNV callers have demonstrated superior precision across multiple bacterial species (23). These computational approaches are essential for distinguishing true mutations from sequencing artifacts, particularly in hypermutator strains or mixed infections.

## 3 Mechanisms of mutations

A mutation is an alteration in the sequence of nucleotide of a brief section of a genome, and the position and intensity of the mutation might affect the phenotypic outcomes. Mutations can be caused by interaction with mutagens, mistakes made throughout DNA replication, or natural mutations. Random, spontaneous alterations to an organism's DNA that take place without the help of outside mutagens are known as natural mutations. They are the starting point for evolutionary change via the introduction of novel genetic variants that are subsequently acted upon by random selection. They are caused by mistakes made during DNA replication, uncontrolled DNA breakdown, or cellular metabolism. Random variance in populations is a result of accidental mutations, which happen at a frequency of 1 X 10^5^ to 10^8^ (24). As bacteria undergo limited genetic recombination and most of their genes are haploid, single mutations can cause phenotypic diversity rather quickly. Mutations may result in altered colony or structural traits or decreased tolerance to antibiotics. The following are some possible outcomes of mutations: Mutations in auxotrophs cause a vital nutrition pathway to malfunction (22); resistant mutants are able to tolerate the stress of being exposed to inhibitory chemicals or antibiotics due to inherited mutations (25); promoter regions and other sequences associated with regulation are disrupted in regulated mutants (24); constitutive mutants are those that consistently show genes that ordinarily turn on and off, like operons (26).

### 3.1 Spontaneous mutations

The existence of spontaneous mutations was conclusively demonstrated by Luria and Delbrück (1) using their fluctuation test, which showed that bacteriophage-resistant mutants in *Escherichia coli* arise at random times during growth, not as a direct response to phage exposure. Subsequently, Lederberg and Lederberg (27) developed replica plating, a technique that provided direct visual evidence that antibiotic-resistant mutants exist in bacterial populations prior to antibiotic exposure. This work fundamentally established that mutation precedes and is independent of environmental selection—a principle central to understanding bacterial adaptation and resistance evolution. Inaccuracies in DNA replication led to spontaneous mutations, which happen without the need for mutation stimulus. Nucleotides are sometimes impaired, introduced, or removed during DNA Pol III's synthesis of a new thread of DNA (28). Therefore, there will be a point mutation. For instance, mispaired nucleotides will appear to swap one nucleotide for another, resulting in a modified granddaughter DNA strand. This can only take place if the bacteria's DNA replicating mechanism experiences two distinct defects (23).

(1) In the replicating fork, DNA pol III links the initial strand with the wrong complementing nucleotide.
(2) The mispairing's chemical action is insufficient to slow down DNA polymerase's polymerase component, allowing exonuclease to eliminate the mispair.

e.g., *Escherichia coli* investigations reveal that the strand that is last experiences spontaneous mutations 20 times more frequently than the strand in the forefront (28).

There is a process called: tautomerization for change the synthesis of nucleotide base pairs throughout DNA replication. It includes a base adopting a temporary, unusual form that affects its hydrogen bonding, letting it to link with the wrong base and resulting in a permanent DNA mutation. For instance, during replication, thymine experiences keto-enol tautomerization. During the initial replication process, this enol species will stick to guanine predominantly. Because DNA replication is semiconservative, the locus of mutation will contain (1) G-C and (3) A-T base pairs at the final stage of the second replication cycle (114).

Erroneous nucleotides may be added or template nucleotides may be deleted as a result of mistakes in DNA replication. For instance, polymerase slippage is more likely to occur in loci that include a large number of short repetitive nucleotides. The DNA Pol III momentarily separates from the template helix during replication. Along with its freshly synthesized strand, the DNA polymerase may move a few repetitions upstream or downstream of its initial location. Since some nucleotides are duplicated twice whereas others do not, slip strand mispairing can lead to addition/deletion mutations (29). The mutation may cause a frameshift if the repetitions are not in a pair of three:

- The original codon is changed into a different codon that encodes for an identical amino acid by a silent mutation (30).
- When a codon in a sequence changes to code for another amino acid, this is known as a missense mutation (31).
- Non-sense mutation: A mutant stopping nucleotide shortens the protein by replacing a wild-type codon and stopping translation (112).

The form of the substitute amino acid's impact on the ultimate protein output determines how severe the mutation is phenotypically. More precisely, the chemical differences of the altered strand amino acid, non-synonymous amino acid replacements result in significant alterations to protein topologies. Nonetheless, there are built-in defenses against these alterations. Few mutations exhibit phenotypic expression due to the multiplicity of codon translation processes and the presence of non-coding regions (32).

### 3.2 Induced mutations

Mutagens can be biological, chemical, or physical in nature. Most of the time, they impact DNA, which can lead to replication mistakes. SOS is an illustration of a biological reaction to damage to DNA that causes mutagenesis and arrest of the cell cycle. Rec A activates DNA polymerases (II, IV, and V) and recognizes single-stranded DNA to trigger the SOS reaction (33). A number of mutagen types and their ensuing consequences are as follows.

#### 3.2.1 Physical mutagens

Mutagens that are physical involve things like UV exposure and radiation. By forming covalent bonds among neighboring pyrimidine bases, UV light ruins DNA. This pyrimidine dimer prevents translation and replication because it does not conform well to the double helix shape of DNA. However, a deletion mutation is typically the outcome of dimer production. Although the consequences of other irradiation forms may differ based on wavelength and magnitude, deletions and insertions are the most common. Purine dimers don't happen very often (34).

#### 3.2.2 Chemical mutagens

Agents that cause mutations whether directly or indirectly are known as chemical mutagens. A chemical mutagen may destroy a base in DNA so that it may no more couple, change the base's constitution and coupling behavior, or replace a base entirely (35). Among them are substances that react with DNA, like the ones mentioned below.

##### 3.2.2.1 Analog bases

They can integrate into DNA because they share substantial structural similarities with nucleotides. An analog of thymine, 5-bromouracil, for instance, functions as a substrate throughout DNA replication and results in point mutations. The base analog produces a tautomer and couples onto guanine rather than adenine, causing this mis-pairing (36).

##### 3.2.2.2 ROS (reactive oxygen species)

When guanine is attacked by hydroxyl radicals, 8 hydroxy-deoxyguanosine (8-OhdG) is created. This miss-pairs with adenine rather than cytosine, causing a (G to T) conversion throughout replication (37).

##### 3.2.2.3 Agents for deaminating

These substances strip nucleotide bases of their amino groups. A cytosine species (uracil) that couples with adenine and an adenine species that combines with cytosine are produced by deaminating agents. When guanine is deaminated, xanthine is produced, which prevents replication and prevents a mutation (113).

##### 3.2.2.4 Aromatic molecules

Neighboring pyrimidine base pairs may interact with compounds such as ethidium bromide. The helix is somewhat unwound by this contact, which also lengthens the space across neighboring base pairs. This intercalation might result in modifications or additions and disturbs the reading frame when translating (38).

##### 3.2.2.5 Alkylating substances

By inserting alkyl groups, substances such as dimethyl nitrosoguanidine and ethyl methanesulfonate change the nucleotide base. Although the kind and location of the alkylation might change, base incorrect pairing typically results in point mutations. Alkylation, despite this, can result in the creation of crosslinks, which prevents reproduction (39).

Although numerous chemical agents able of inducing mutations have been characterized in laboratory systems, their relative contribution to mutation dynamics in natural microbial environments remains incompletely understood (35, 39). Most experimental studies rely on controlled laboratory conditions that may not fully replicate the complex environmental stresses encountered by pathogenic bacteria in clinical settings. Consequently, further investigations are needed to evaluate how combinations of environmental stressors, host immune responses, and antimicrobial exposure interact to influence mutation frequency and spectrum *in vivo*.

#### 3.2.3 Biological mutagens

DNA from viruses and transposons are examples of natural mediators of mutation. Transposons are regions of DNA that may shift and duplicate spontaneously. Placement of a transposon within a segment of DNA can affect gene functioning. Though mechanically comparable, transposition is not formally a form of recombination. On both sides of the transposition pattern, transposons frequently couple with brief nucleotide repetition segments (40).

### 3.3 Evolutionary significance of mutation bias

The classical view of mutations as random events, established by Luria and Delbrück (1) and supported by Lederberg and Lederberg's (27) replica plating experiments, held that mutations occur without regard to their potential benefit. However, recent advances in evolutionary microbiology have challenged the traditional view of mutations as purely random events. In evolutionary microbiology, the term “random” has been used in two distinct senses, which are often conflated. First, in the classical sense established by Luria and Delbrück (1), mutations are said to be **random with respect to fitness**—meaning they occur without regard to whether their effect will be beneficial, neutral, or harmful to the organism at that moment. Organisms do not ‘direct' mutations to genes that would confer a selective advantage. This remains the accepted definition among evolutionary biologists.

Second, the **distribution of mutations** across the genome is not uniform. Some genomic regions.

(e.g., mutational hotspots, regions near the replication terminus) and certain nucleotide changes (e.g., transition biases) occur at higher frequencies than others. This phenomenon is known as **mutation bias**. Importantly, mutation bias does **not** contradict the classical definition of randomness with respect to fitness. Mutations are still undirected in their adaptive value, but their probability of occurrence is unevenly distributed across sites and mutation types.

Recent advances have demonstrated that mutation bias significantly shapes adaptive trajectories in bacterial populations (15). Mutation bias can either constrain or facilitate adaptation depending on environmental context. This represents a refinement—rather than a rejection—of classical mutation theory, moving toward a more nuanced understanding where the probability of specific adaptive outcomes is influenced by underlying mutation rates. As highlighted by Cano et al. (11) and Sane et al. (12), adaptive mutations in *Mycobacterium tuberculosis* and *Escherichia coli* follow predictable biased mutational spectra, yet these mutations still arise without foresight of their benefit.

The clinical relevance of this bias is particularly evident in antibiotic resistance development.

Cano et al. (11) demonstrated that adaptive mutations in *Mycobacterium tuberculosis* and *Escherichia coli* consistently follow predictable mutational spectra, suggesting that resistance evolution may be more predictable than previously appreciated. This has profound implications for antimicrobial stewardship, as understanding mutation bias could enable proactive rather than reactive therapeutic strategies.

Furthermore, the relationship between mutation rate and evolutionary success involves a delicate trade-off. While hypermutator phenotypes accelerate adaptation by generating diverse genetic variants, they also accumulate deleterious mutations that reduce overall fitness. Sane et al. (12) elegantly demonstrated that shifts in mutation spectra enhance access to beneficial mutations while minimizing fitness costs, revealing sophisticated evolutionary optimization at the molecular level.

Despite growing evidence supporting the influence of mutation bias on evolutionary trajectories, the extent to which mutation bias vs. natural selection shapes adaptive outcomes remain an area of active debate. Some researchers argue that selective pressures exert a stronger influence on evolutionary direction, whereas others emphasize the role of intrinsic mutational biases in determining which genetic variants arise in the first place. Resolving this debate requires integrative studies that combine experimental evolution, genomic analysis, and mathematical modeling (11, 12).

### 3.4 Unresolved controversies and limitations in mutation mechanism studies

Despite decades of research, several controversies regarding mutation mechanisms remain unresolved. First, the relative contribution of **spontaneous vs. induced mutations** in natural environments is unclear. Most studies quantify mutation rates under laboratory conditions (e.g., rich media, 37 °C), which poorly replicate the nutritional and oxidative stresses of host tissues or soil ecosystems (41). Conflicting findings exist: some reports suggest that oxidative damage dominates *in vivo* mutation spectra, while others implicate replication errors as the primary source (23, 42).

Second, the **transient hypermutator phenotype**—where bacteria temporarily elevate mutation rates under stress via SOS response—is well documented *in vitro*, but its prevalence and duration in chronic infections remain unknown. Clinical isolates often show fixed mutator genotypes (e.g., MMR defects), yet whether transient hypermutation precedes or substitutes for stable mutators is debated (22).

Third, **reproducibility concerns** arise from strain-specific mutation rates. Even within *E. coli*, different laboratory strains (K-12 vs. B vs. clinical isolates) exhibit up to 10-fold variation in spontaneous mutation rates due to differences in repair gene polymorphisms. This complicates cross-study comparisons and meta-analyses.

**Limitation of current models:** Most mutation studies use exponentially growing planktonic cultures, ignoring biofilm physiology, where slow growth and spatial structure dramatically alter mutation frequencies and spectra. Biofilm persister cells, in particular, may represent an underexplored reservoir for mutation accumulation (43).

### 3.5 An evolutionary framework for mutation: fitness landscapes, epistasis, and contingency

Drug resistance and mutagenesis are fundamentally evolutionary processes. Understanding them requires moving beyond mechanistic descriptions of mutation origins to a population and molecular evolutionary perspective. Key concepts from evolutionary biology—fitness landscapes, epistasis, clonal interference, convergent evolution, adaptive trade-offs, mutational robustness, and contingency—provide the necessary framework.

#### 3.5.1 Fitness landscapes and seascapes

A **fitness landscape** maps all possible genotypes (or mutations) to their reproductive success in a given environment. In the context of antibiotic resistance, bacteria must navigate this landscape where peaks represent high resistance or high growth rates, and valleys represent fitness costs. The work of Flynn et al. (44) empirically illuminated fitness landscapes for the essential chaperone Hsp90, quantifying how hundreds of missense mutations affect cellular fitness and revealing that a protein's tolerance to mutation is highly context-dependent.

Because clinical environments change (e.g., fluctuating drug concentrations), the landscape is better described as a **fitness seascape**. This dynamic view is essential for understanding trade-offs: a mutation that confers high resistance under drug pressure may impose severe growth defects when the drug is removed (45).

#### 3.5.2 Epistasis: genetic background alters mutation effects

The fitness effect of a mutation is not fixed; it depends on other mutations present in the genome. This phenomenon is **epistasis**. Two forms are particularly relevant to resistance evolution:

- **Diminishing-returns epistasis:** As bacteria accumulate beneficial mutations, each additional mutation provides a smaller fitness gain (46).
- **Sign epistasis:** A mutation may be beneficial in one genetic background but deleterious in another. This creates rugged fitness landscapes where evolutionary trajectories can be trapped on local peaks.

Importantly, the strength of epistasis varies with antibiotic concentration. Under high drug pressure, the strong selective advantage of resistance mutations can smooth the landscape, making evolution **more predictable** than in drug-free conditions (47).

#### 3.5.3 Clonal interference and convergent evolution

In large bacterial populations, multiple beneficial mutations often arise simultaneously in different lineages and then compete. This **clonal interference** slows the rate of adaptation and can cause the loss of otherwise beneficial mutations (48). The effect is amplified in clinical settings where population sizes are enormous.

When the same resistance mutations arise independently in different populations, this is **convergent evolution**. Under strong, constant antibiotic selection, convergence is common. However, sequential treatment with multiple drugs can shift evolution from divergence to **convergence** by repeatedly purging resistance mutations that cause collateral sensitivity, forcing multidrug resistance to evolve along similar pathways (49).

#### 3.5.4 Fitness trade-offs and compensatory evolution

Resistance mutations almost always carry a **fitness cost** in the absence of antibiotics—slower growth, reduced metabolic efficiency, or increased susceptibility to host immune defenses. This trade-off is the basis for “collateral sensitivity” strategies.

However, bacteria can overcome these costs through **compensatory evolution**: secondary mutations that restore fitness without reversing the original resistance mutation. This explains how highly resistant strains persist and dominate even after antibiotic cessation (50). Compensatory mutations often occur in regulatory genes or in the very same protein as the resistance mutation, restoring structural stability.

#### 3.5.5 Mutational robustness and evolvability

Some genetic architectures buffer the harmful effects of mutations, allowing genetic variation to accumulate in a “hidden” form and later be revealed under stress. This property is **mutational robustness**.

A classic example is the chaperone protein **DnaK (Hsp70)**, which assists protein folding. By suppressing the deleterious effects of many mutations, DnaK acts as a **capacitor for evolution** (51). Interestingly, (52) demonstrated that Hsp90 has both general and client-specific buffering activities, meaning that a single protein can disproportionately control the evolvability of entire pathways.

#### 3.5.6 Contingency, chance, and the unpredictability of evolution

The work by Park et al. (53) has fundamentally challenged deterministic views of evolution. By reconstructing ancient proteins and engineering all possible mutations, Thornton's laboratory demonstrated **epistatic drift**: as a protein evolves, the fitness effect of any given mutation changes over time, often in a random and unpredictable manner.

This means that the fate of a mutation is shaped not only by natural selection but also by the **chain of chance events** in its evolutionary past. Consequently, evolutionary history is deeply **contingent**, and “rewinding the tape of life” would likely produce different outcomes each time (54). For drug resistance, this implies that even under identical antibiotic pressures, different bacterial lineages may evolve resistance via different mutational paths—a finding with profound implications for predicting resistance.

#### 3.5.7 Mutagenesis as a contextual process

Mutation rates and spectra are not fixed properties of a genome; they are highly dynamic and context-dependent. Under stress (e.g., antibiotic exposure), bacteria activate the **SOS response**, upregulating error-prone DNA polymerases that actively increase mutation rates in a targeted, transient manner (22). Intracellular metabolites, such as reactive oxygen species generated during aerobic metabolism, are potent mutagens, linking central metabolism to mutation frequency (42). Furthermore, **persister cells**—dormant subpopulations that survive antibiotic treatment—serve as critical reservoirs for future mutations. When they regrow after treatment ends, they provide the raw material for the evolution of stable, heritable resistance (43). Thus, mutagenesis is not a passive background process but a regulated, environmentally responsive driver of bacterial adaptation.

## 4 Virulence factors and mutations

### 4.1 Conflicting findings and knowledge gaps in virulence gene mutations

The relationship between mutation and virulence is not straightforward. Conflicting reports exist regarding whether virulence factor mutations generally increase, decrease, or have context-dependent effects on pathogenicity. For example, mutations in the *mprF* gene of *S. aureus* can simultaneously reduce daptomycin resistance (beneficial for treatment) but also alter membrane charge, affecting immune recognition—with conflicting outcomes depending on the infection model (mouse vs. human serum) (55).

Unresolved controversy: Do compensatory mutations that restore fitness costs of resistance also restore virulence? Some studies show that resistant mutants with fitness-compensating mutations regain full virulence, while others report a trade-off where resistance stabilization reduces toxin production. This controversy has direct clinical implications: if resistance evolution consistently attenuates virulence, then aggressive antibiotic treatment might inadvertently select for less dangerous strains—but the evidence is mixed (56).

Reproducibility concern: Virulence assays are notoriously variable across laboratories due to differences in animal models (mouse strain, infection route, inoculum size), making meta-analysis difficult. Many published mutation-virulence associations fail to replicate in independent host backgrounds. The field would benefit from standardized *in vivo* evolution protocols (56).

**Figure 2:**
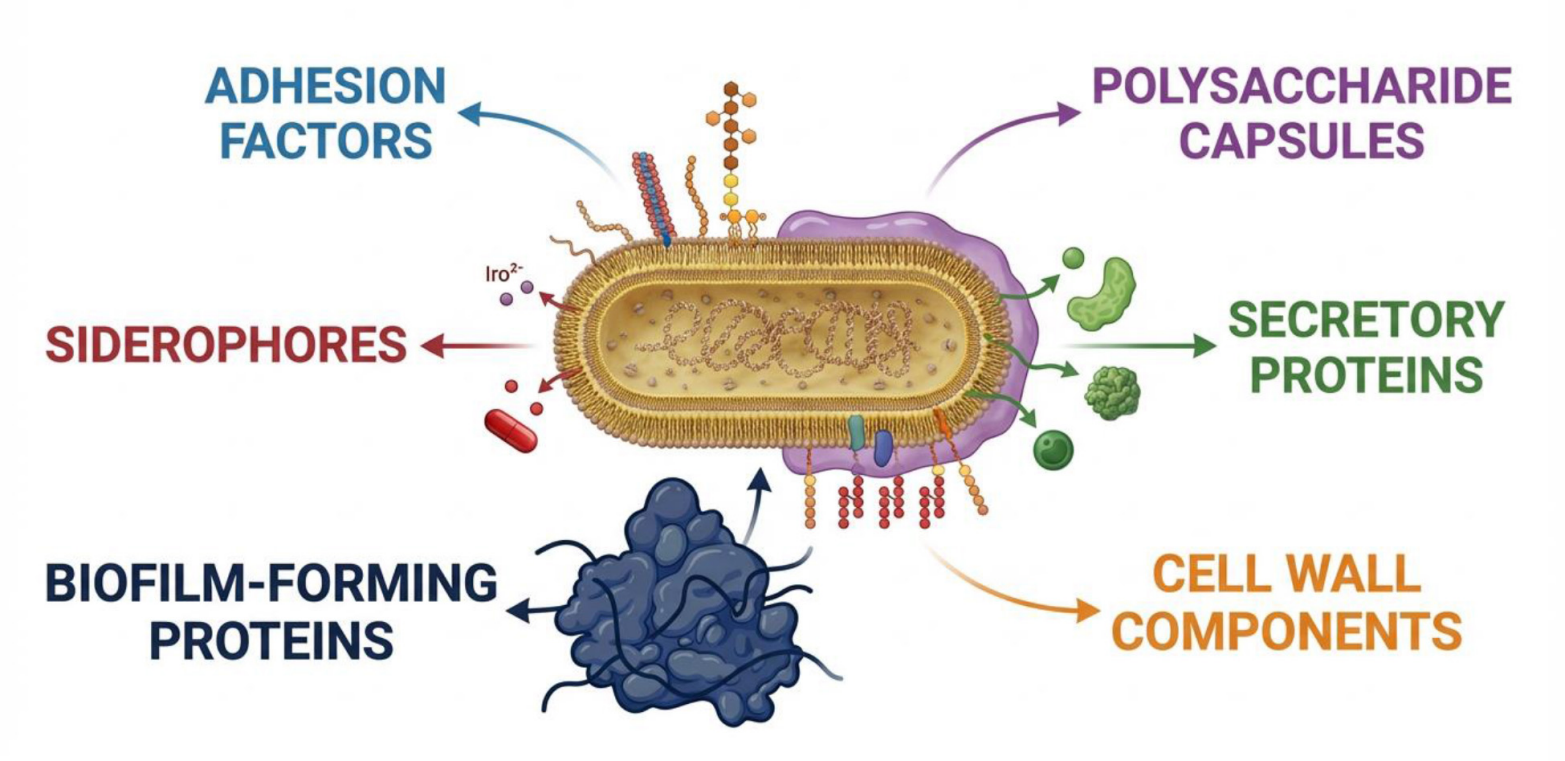
The six principal categories of bacterial virulence factors classified by their functional roles in pathogenesis. (1) Adhesion factors (pili, fimbriae, adhesins) mediate host cell attachment, while (2) polysaccharide capsules provide antiphagocytic protection. (3) Secretory proteins, including toxins and enzymes, modify the host cellular environment, and (4) cell wall components (LPS, lipoteichoic acids) trigger inflammatory responses. (5) Biofilm-forming proteins enable community-based resistance, and (6) siderophores facilitate iron acquisition from host proteins.

Despite improvements in detection and therapy, bacterial infections continue to be a serious global public health concern. It is essential to comprehend how pathogenic microorganisms communicate with the host to cause clinical illness. Finding novel virulence factors that could be targeted for vaccine and drug discovery is a crucial initial phase in this approach. Several kinds of bacterial virulence factors (Figure 2) can be distinguished based on their intended role and virulence mode (20). (1) One of these membrane proteins is involved in adhesion, colonization, and invasion; it also facilitates attachment to host cell exteriors, causes resistance to medications, and facilitates interaction between cells. (2) The bacterial cell is encased in polysaccharide capsules that possess antiphagocytic characteristics. (3) Several interactions between bacteria and host cells are caused by secretory proteins, like toxins, which can alter the surroundings of the host cell (57).

Different systems for secretion are employed by bacterial pathogens, most notably types I–IV (58). To move protein poisons into the extracellular structure or host from their cytoplasm (59). Numerous pathogenic Gram-negative bacteria use autotransporters (ATs), which are virulence proteins, to move from the cell membrane to the extracellular milieu or cell surface. The type V pathway sequentially secretes a class of proteins known as ATs (60). Oher researchers have studied the structure and suggested mechanism of ATs (61). (4) Elements of the cell wall and external membrane including lipoteichoic acids and lipopolysaccharide. Gram-negative bacteria can fend off complement-mediated lysis owing to the main exterior glycolipid, LPS, whereas Gram-positive bacteria are inherently encased in a dense cell wall with minimal porosity to their surroundings. LPS is a strong inflammatory inducer and triggers the host complementing cascade (62). (5) Additional virulence factors, include siderophores and proteins that produce biofilms (Figure 2). Certain bacteria, including *Streptococcus pneumoniae, Pseudomonas aeruginosa*, and *Staphylococcus aureus*, can produce biofilms (63). The development of biofilms gives bacteria a selective edge in terms of their natural durability and resistance to antibiotics. It also makes it easier for the bacteria to colonize the host. Furthermore, several bacterial virulence genes interfere with normal host cell functions by mimicking human proteins (64).

Defining virulence factors is vital in studying bacterial pathogenicity and their relationships with the place of residence, which potentially also act as innovative candidates in therapy and vaccine discovery. In the pregenomic period, comprehensive characterization of virulence factors was usually done either by biochemical techniques or via genetic screens for genes generated during *in vivo* settings or essential for surviving in the recipient (e.g., Signature-Tagged Mutagenesis). Over the past few decades, the investigation of virulence factors has been hastened by the advent of post-genomic techniques. Candidate virulence genes are quickly added to databases by bacterial sequencing of genomes. The ever-changing “transcriptomic” and “proteomic” investigations, often known as functioning genomic research, go in addition to this somewhat static depiction of the cell (64).

The most widely used method for studying harmful bacteria is still gel-based proteomics, despite the development of numerous other approaches. The ability to examine post-translational protein changes that might not be visible from the examination of nucleotide sequencing information is a notable benefit of proteomics above genomics. Since it has been shown that post-translational protein changes are crucial for virulence factors, attempts have been made to determine how they contribute to bacterial pathogenesis utilizing proteomic approaches. Determining the proteomes from various cellular compartments, particularly the cell surface, is another use of proteomic techniques. Determining particular functions and impact of virulence factor methods of disease and how they communicate with host cells is another challenge once proteomics has determined them (64).

Several historical studies (Table 1) have detected mutations in virulence genes of bacteria (64). The comprehensive proteomic examination of detergent-derived outer membrane vesicles (DOMVS) from the New Zealand epidemic strain has been published by Ferrari et al. (65), who contrasted the proteome of group B *N. meningitides* with its counterpart in the outer membrane vesicles of *N. meningitidis* delta gna33 mutant, where the gene responsible for a lytic transglycosylase was eliminated. This research remarkable shows that precise choosing of particular mutations is an efficient method of obtaining highly enriched membranes that indicate an intriguing potential vaccine to DOMVs. Besides, the roles of glycosyltransferases—*PglA, PglC*, and *PglJ*—involved in the manufacture of the *Campylobacter jejuni* N-linked heptasaccharide glycan was first directly demonstrated by Linton et al. (66) using mutational and MS/MS studies. The function-based variation of the AT superfamily is surely influenced by the significant variability observed in the post-translational editing of constituent domains in ATs, including lipidation, glycosylation, oligomerization, and dissociation by a range of processes (61).

**Table 1:** Examples of bacterial and host mutations upon infection by pathogenic bacteria.

Target	Alteration	Function	Bacterial name	Reference
*h3S10*	De-phosphorylation	Immune regulation	*L. monocytogenes*	Hamon et al. (98)
*h3K4*	Methylation	Transcription activation of ribosomal gene	*L. pneumophila*	Li et al. (99)
*h4*	deacetylation	Interferon-γ-dependent HLA-DR gene expression	*M. tuberculosis*	Wang et al. (100)
*p300*	Repression	reduced IL-8 production	*E. coli*	Shames et al. (101)
*hDAC2*	Repression	Gene activations	*P. gingivalis*	Aruni et al. (102)
*hKMT*	Repression	Downregualtion of heterochromatin marks	*P. gingivalis*	Aruni et al. (103)
*mad2L2/mAD2B*	Cell cycle	APC/Ccdh1 Inhibition	*S. flexneri*	Iwai et al. (104)
*cRL*	Cellular proliferation	Inhibition of G2/M phase	*B. pseudomallei*	Jubelin et al. (105)
*pglC, pglA, and pglJ*	In activation	N-linked glycan biosynthesis pathway	*Campylobacter concisus*	Arbour et al. (106)

## 5 Drug resistance and mutations in bacteria

The concept that mutations conferring resistance arise spontaneously prior to antibiotic exposure was elegantly demonstrated by Lederberg and Lederberg (27), who showed that streptomycin-resistant colonies of *E. coli* could be isolated without ever exposing the bacteria to streptomycin, using their replica plating technique. Additionally, the bacteria may undergo mutations in their individual chromosomal DNA. Either an interim or perpetual transfer may be made. GyrA mutations, which affect DNA gyrase and impart fluoroquinolone resistance, and rpoB mutations, which modify RNA polymerase and confer rifampin resistance, are two examples of chromosomal mutations that confer drug resistance. Mutations in 23S rRNA genes that inhibit the binding of oxazolidinone (such as linezolid) or rpsL mutations that alter the streptomycin-binding site on ribosomes are further examples (67, 68). Bacteriophage-borne transfer of genes for resistance is rather uncommon; plasmid-mediated transfer is the more frequent method of acquiring external genetic material. Some bacteria, like *Acinetobacter* species, are able of absorbing genetic information from their surroundings because they are intrinsically competent. Genetic material and external stresses, such as nutrient shortages, UV rays, and antimicrobial consumption, can be internalized or sensed by bacterial surface and structural components such as integrins and insertion repeats, which have been linked in several strains to regulating mechanisms related to antibiotic resistance acquisition. One mutation occurs in bacteria on an average for each 1X10^6^ to 1X10^9^ division of cells, and the majority of these mutations are harmful to the cell (69). Genes generating drug targets, drug carriers, controllers that regulate drug carriers, and antibiotic-modifying enzymes are typically the sole gene groups that experience mutations that contribute to resistance to antibiotics (70). Furthermore, the organism incurs costs as a result of numerous changes that provide resistance to antimicrobial agents. For instance, the development rate of *Staphylococcus aureus* is considerably reduced when the organism develops resistance to methicillin (19). Mobile genetic elements (MGEs) like plasmids and transposon elements are used in conjugation to transfer important genetic information for the evolution of bacteria. High mutation rates are caused by certain bacteria acquiring mutations in DNA repair genes. The activation of error-prone SOS-response DNA polymerases and mutations in the mismatch repair (MMR) system, which increase DNA replication mistakes, are two examples of abnormalities in DNA repair or error avoidance that cause bacterial hypermutation. These hypermutable states can be directed to particular DNA regions, are frequently temporary, and are brought on by environmental stressors like antibiotics. Hypermutation accelerates the pace of advantageous mutations for adaptation, but it also produces a large number of harmful ones, with the advantageous ones being gradually filtered out (22). Furthermore, the horizontal gene transfer (HGT) of antibiotic-resistant genetic material (ARGs) across bacteria is facilitated by bacterial outer membranes vesicles (OMVs). Gram-negative bacteria produce these nanostructures, which serve as organic transporters containing and transfer DNA, particularly ARGs, to other bacteria, thereby aiding in the development of multidrug resistance (22, 26). The fact that using these medications increases resistance is one of the major challenges of antimicrobial resistance. High-level resistance in subsequent bacterial generations can be selected for by using sub-inhibitory antimicrobials at low or very low levels. Additionally, hypermutatable strains of bacteria may be selected for increasing the ability to develop opposition to other antimicrobial substances, and promoting the transmission of genetic components that are mobile (71).

**Figure 3:**
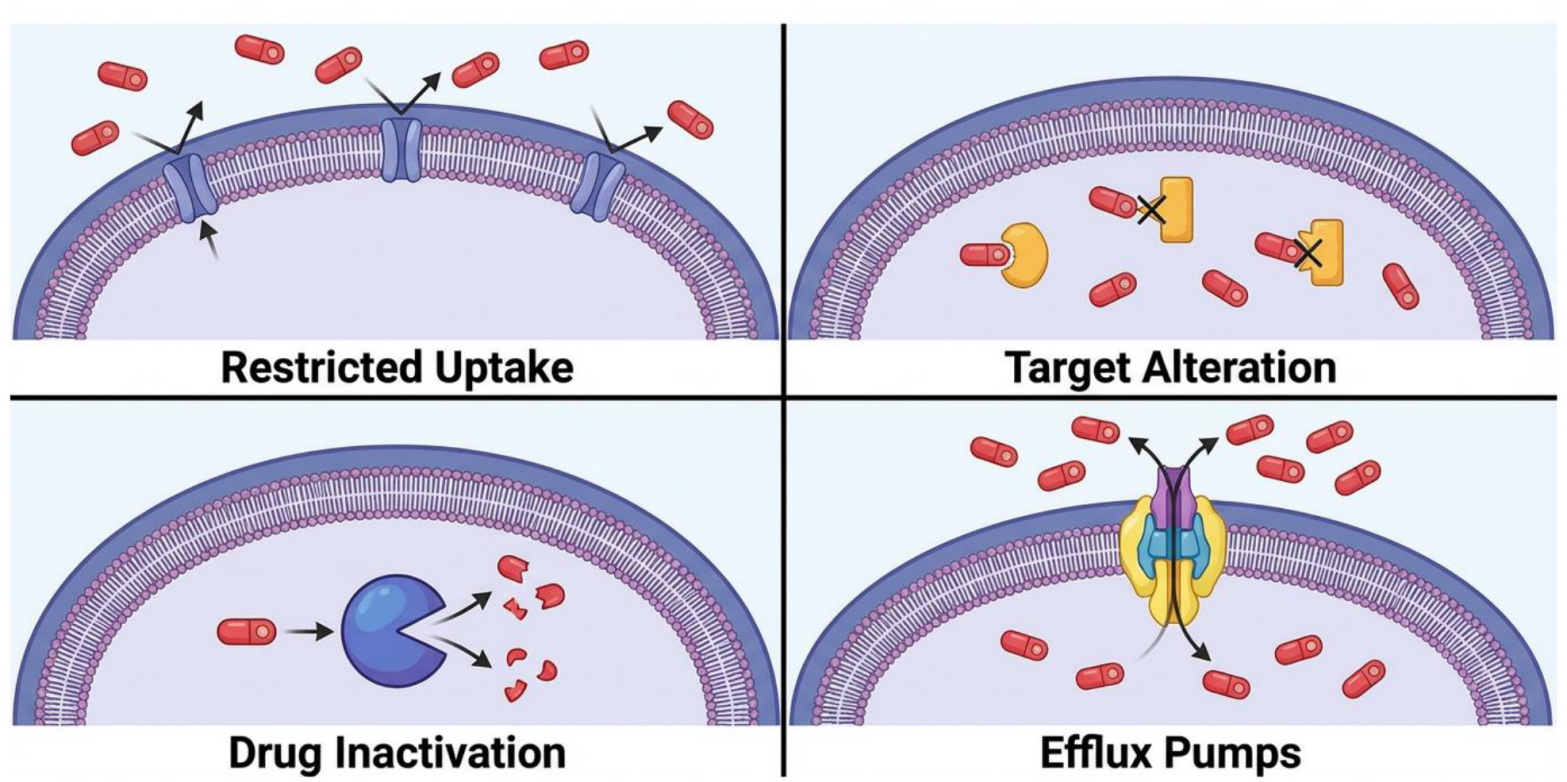
Four major pathways of antimicrobial resistance in bacteria: restricted uptake, target alteration, drug inactivation, and active efflux.

The four main types of antimicrobial resistance pathways are: (1) restricting medication uptake; (2) altering a drug target; (3) rendering a drug inactive; and (4) regulating drug efflux (Figure 3). Restricted uptake, drug suppression, and drug efflux are examples of intrinsic resistance processes; drug targeting alteration, drug deactivation, and drug efflux are examples of developed resistance strategies. Gram negative bacteria and Gram-positive bacteria employ different kinds of processes due to structural variations. While, some of Gram-positive bacteria lack the ability to use some drug efflux mechanisms and less frequently use restricting drug intake, Gram negative bacteria employ all four major pathways (21).

It is well known that certain strains of the Enterobacter can develop resistance as a result of decreasing the quantity of porins and occasionally ceasing to produce some porins altogether. These bacteria decrease the quantity of porins as a defense against carbapenems (72). Some bacteria have been observed to exhibit mutations that alter the porin channel, resulting in resistance to imipenem as well as tetracycline and β-lactam antibiotics (73). On the other hand, Vancomycin resistance was has grown to be a significant problem in Methicillin-resistant *Staphylococcus aureus* (MRSA) and enterococci. The mechanism of tolerance is the acquisition of *van* genes, which alters the composition of precursors to peptidoglycan and reduces vancomycin's ability to binding (56). Gene mutations (e.g., *mprF*) cause the cell outermost membrane to become positively charged, which prevents calcium and, consequently, daptomycin from adhering where calcium must be present for daptomycin to attach to cells (55).

Ribosomal alterations, ribosomal subunit methylation, which most frequently involves *erm* genes, or ribosomal defense are the three ways that resistance to medications that target the ribosomal subunits can develop. These processes prevent the medication from attaching itself to the ribosome. These processes vary widely in the degree of drug interaction (74). Besides, resistance to medications that block metabolic processes is caused by either an excess of resistant enzymes or mutations in the enzymes associated with the folate biosynthesis pathway. Since the sulfonamides and trimethoprim are structural analogs of the natural substrates, they bind to their corresponding enzymes. These medications work by attaching to the enzymes' active sites and causing competitive inhibition. These enzymes typically have mutations in or close to the active region, which alters the structure of the enzyme and prevents drug binding while permitting the substrate that is natural to attach (19).

According to our systematic literature search, we identified approximately 846 studies focusing on mutations in virulence and drug-resistant genes in bacteria, with an accelerating rate of recent publications. Table 2 summarizes some of the most recent mutations in virulence and resistance genes in pathogenic bacteria.

**Table 2:** Target genes for mutations in drug resistance and virulence genes in pathogenic bacteria in some recent studies.

Target	Alteration	Function	Bacterial name	Reference
*pirA; piuA; piuC*	Siderophores alterations	Limited drug uptake	*P. aeruginosa*	Viñes et al. (75)
*fhuA, iutA, cirA, sitC, apbC*,	Siderophores alterations	Limited drug uptake	*K. pneumoniae*	Padovani et al. (107)
*hisJ*	Include susceptibility to host-derived AMPs	Modify drug target	*P. aeruginosa*	Hernández-García et al. (108)
*3 ftsI*	Peptidoglycan synthesis	Drug efflux	*P. aeruginosa*	Rapsinski et al. (76)
*mexB*	Substitution of C or G	Drug efflux	*P. aeruginosa*	Yamasaki et al. (109)
*mcrAB*	Unaltered acrB expression	Drug efflux	*E. coli*	Nasralddin et al. (77)
*nt5*	Alteration in cell membrane integrity	Inactivation of drug	*S. aureus*	Liu et al. (92)
*cTX-M*	decreased susceptibility	Beta-Lactamase	*E. coli*	Tellapragada et al. (110)
rpoB	Changes affecting Ser-531, His526, and Asp-516 codon	Build the RNA polymerase complex,	*Mycobacterium tuberculosis*	Li et al. (37)
gyrA	Encodes the DNA gyrase A subunit	pPovides instructions for making the GyrA subunit of DNA gyrase	*K. pneumoniae*	Rezaei et al. (111)

### 5.1 Clinical implications of mutation-driven resistance

The translation of mutation mechanisms into clinical practice requires understanding how *in vitro* observations correlate with *in vivo* outcomes. Seif et al. (25) comprehensively mapped auxotrophic mutations in Gram-negative pathogens, revealing that metabolic deficiencies created by resistance-conferring mutations can be therapeutically exploited. This concept of “collateral sensitivity” has gained traction as a potential strategy to steer evolution toward clinically manageable outcomes.

Recent clinical surveillance studies have identified concerning trends in mutation-mediated resistance. Viñes et al. (75) documented novel *PiuC, PirA*, and *PiuA* mutations leading to *in vivo* cefiderocol resistance progression in *Pseudomonas aeruginosa* from a leukemic patient, highlighting how individual patient microbiomes can evolve resistance during treatment. Similarly, Rapsinski et al. (76) demonstrated that mutations during *in vivo* exposure to ceftazidime/avibactam subsequently conferred resistance to ceftolozane/tazobactam and imipenem/cilastatin/relebactam, revealing unexpected cross-resistance patterns that complicate sequential antibiotic therapy.

The clinical challenge is compounded by heteroresistance, where subpopulations with different resistance levels coexist. Nasralddin et al. (77) showed that tetracycline and chloramphenicol exposure induces decreased susceptibility to tigecycline through genetic alterations in AcrABTolC efflux pump regulators, demonstrating how exposure to one antibiotic can inadvertently select for resistance to structurally unrelated drugs.

### 5.2 Conflicting findings and model limitations in resistance mutation research

**Conflicting finding #1: The cost of resistance:** While it is widely accepted that resistance mutations incur fitness costs in the absence of antibiotics, recent studies show that many clinical isolates carry resistance mutations with **negligible or no fitness cost**—sometimes even conferring a fitness advantage in drug-free conditions (68). This contradicts earlier models that assumed resistance inevitably carries a trade-off. The discrepancy arises from differences in genetic background, compensatory evolution, and the specific resistance mechanism (efflux vs. target modification).

**Conflicting finding #2: Mutation bias vs. selection:** Cano et al. (11) argue that mutation bias dominates the spectrum of adaptive resistance mutations, while others contend that selection is the primary filter, and bias plays a minor role. Resolving this requires distinguishing between *available* mutations (determined by bias) and *fixed* mutations (determined by selection). The field lacks a consensus quantitative framework.

**Limitations of current models:** Most resistance evolution studies use fixed antibiotic concentrations in liquid culture, whereas clinical exposure involves fluctuating, sub-inhibitory, and spatially heterogeneous concentrations. New microfluidic and *in vivo* imaging models reveal that spatial structure and gradients dramatically alter mutation selection dynamics, but these models are not yet standardized.

**Reproducibility concern:** The widely cited phenomenon of “collateral sensitivity” (resistance to one antibiotic increases sensitivity to another) shows poor reproducibility across bacterial strains and growth conditions. Some laboratories report strong collateral effects for specific drug pairs (e.g., tetracycline-chloramphenicol), while others find no effect or even cross-resistance (77).

## 6 Reproducibility, limitations, and unresolved controversies across the field

### 6.1 Reproducibility of mutation studies

Several factors undermine reproducibility in bacterial mutation research. First, **mutation rate measurements** vary widely depending on the method used (fluctuation test, Luria-Delbrück, mutant accumulation, sequencing). The same strain can yield 5- to 10-fold different rates across laboratories due to differences in plate counting, media, and statistical correction methods.

Second, **strain background effects** are often underreported; a mutation studied in *E. coli* MG1655 may not behave identically in a pathogenic isolate. Third, **culture history** (e.g., prior freeze-thaw cycles, number of passages) alters mutation spectra through unknown mechanisms.

### 6.2 Limitations of current models

***In vitro* vs**. ***in vivo*:** Laboratory evolution in rich broth selects for different mutations than those arising in host environments (e.g., limited iron, immune pressure). The translation from test tube to clinic remains poor. Also, **Single-species vs. polymicrobial:** Most studies use pure cultures, but infections involve multispecies communities where metabolic interactions alter mutation rates. For example, co-culture with *P. aeruginosa* can increase mutation rates in *S. aureus* via oxidative stress—a finding rarely incorporated into resistance models. In addition to **Short-term vs. long-term evolution:** Most experiments run for days to weeks, yet clinical resistance emerges over months of treatment. Long-term (>500 generations) dynamics reveal different mutation trajectories, including plateau effects and reversions, which are missed in short assays.

### 6.3 Unresolved controversies

1. **Are mutator strains beneficial or detrimental to bacterial populations in the long term?** Theoretical models predict that mutators are selected during periods of stress but are outcompeted by wild-type strains when stress subsides. However, clinical isolates frequently carry stable mutator alleles (e.g., *mutS, mutL* defects), suggesting that chronic infection environments maintain selection for mutators indefinitely. This remains unresolved.
2. **Does mutation bias actually constrain adaptation, or do compensatory mutations overcome bias?** While bias makes certain mutations more likely, bacteria can acquire compensatory mutations elsewhere that achieve similar adaptive outcomes via different pathways. The degree of “evolutionary repeatability” is hotly debated.
3. **Persister cells: a distinct mutational reservoir or an experimental artifact?** Some argue that persisters are a genuine subpopulation with altered mutation rates; others contend they are simply slow-growing cells that do not accumulate mutations differently. Single-cell sequencing could resolve this, but technical challenges remain.

## 7 Interaction between mutant and non-mutant bacteria

The efforts to link between the genotype and phenotype of microbial species and their potential to interact for the purpose to better understand bacterial etiology or intricate problems of flexible feasible, pathogenic, or biochemical significance have placed an inconvenience on the methods needed for identification and grouping of non-mutant and mutant bacteria. In order to identify gene functions, track the evolution of resistance, and observe the impacts of natural selection on bacterial populations, it is crucial to compare mutant and non-mutant bacteria while investigating mutant genes by comparing the genetic composition and observed characteristics of the two types (15). Mutations that are of significant impact in bioengineering because they can result in the regulated development of proteins with altered or improved functions. In broad terms, methods for testing for bacterial mutants depend on examining each mutant's phenotype independently, either using an antibiotic picking marker, measuring the enzyme activity in cell lysates, or on dishes with the proper enzymatic bases (such as X-Gal evaluation for the level of reporter enzyme activity) (78).

In addition, there are numerous interactions among the different kinds and shapes of bacteria in the ecological system. Phycospheres are inhabited by a variety of bacteria that can profit from the metabolites of phytoplankton, usually belonging to the Bacteroidetes and Alphaproteobacteria (79). The bacterial species that inhabit phycospheres probably compete with one another for resources such as vital metals, nutrient compounds, and molecules (80, 81). The overwhelming presence of copiotrophic bacteria—bacteria with big, tightly controlled genomes and the ability to expand quickly under the right circumstances— in phycosphere populations has been attributed to these competitive relationships (82). The connections between phycosphere bacteria take place at the micron scale, yet ecosystem connections between them have the capacity to affect carbon flux rates and effectiveness at a worldwide level. When bacteria rates of development and levels of substrate in laboratory settings are far higher than those found in natural environments, it might be difficult to identify genuine bacterial–bacteria interactions (41).

The connections, whereby a particular organism gains from another's metabolic processes. For instance, an intended recipient strain that uses a metabolic by-product generated by one genotype may benefit from it (83). Since the liberated metabolite has no costs to its creator and the recipient gains by impulsively using this resource, this straightforward form of facultative cross-feeding is straightforward to comprehend from the perspective of evolution (84). In contrast, metabolic connections occur when two or more bacteria join forces to carry out expensive biochemical tasks that are impossible for either one of them to do on their own. Bacterial forms typically engage in this kind of cooperative interaction, which frequently relies on the mutually beneficial transfer of certain substances (85). Characteristics like the development of physical adherence components (86) or the depletion of vital biosynthetic runs (87) have been documented in cases where the communication participants have been examined in greater detail. These features may have emerged as specific modifications to the symbiotic behavior. Other researches concentrate on interactions during feeding (88). According to (116), recent methods such as metagenomics could describe these interactions between different kinds of microorganisms in different habitats.

The application of bioinformatics techniques has improved our knowledge of life processes and led to the creation of new statistical metrics and algorithms that evaluate the links between the members of big data sets. *In silico* computational methods have advanced quickly, making such analysis possible. Databases focusing on various stages of virulence of bacteria and their interactions, for example, can be created by conducting research on and interpreting of nucleotide and peptide sequences, protein regions, and structures for proteins (89). Using bioinformatics, a number of recent research investigations have identified genes that encode infectiousness traits, fitness, metabolic processes, interactions between bacterial mutant and nonmutant strains, and molecules of focus for possible vaccines toward infectious bacteria like S. aureus (90, 91).

## 8 Futures of mutations

### 8.1 Emerging technologies for studying mutation dynamics

The past five years have witnessed revolutionary advances in our ability to study bacterial mutations at unprecedented resolution. Single-cell sequencing technologies now enable detection of rare mutational events within heterogeneous populations, revealing that mutant subpopulations can arise and expand much faster than previously recognized (22).

CRISPR-based approaches have transformed functional genomics in bacteriology. Al-Fadhli and Jamal (17) comprehensively reviewed recent advances in gene-editing approaches for tackling antibiotic resistance, highlighting how CRISPR-Cas9 can precisely target and eliminate resistanceconferring genes or selectively sensitize resistant bacteria to conventional antibiotics. Liu et al. (92) demonstrated that *Staphylococcus aureus nt5* gene mutation through CRISPR RNA-guided base editing weakens bacterial virulence and immune evasion, establishing a proof-of-concept for therapeutic editing.

Machine learning applications are now being deployed to predict mutation outcomes. By training algorithms on large-scale mutational datasets, researchers can now forecast which mutations are likely to emerge under specific selective pressures with remarkable accuracy (7). These predictive models are being integrated into clinical decision support systems to guide empiric antibiotic therapy based on predicted resistance evolution.

High-throughput experimental evolution platforms coupled with whole-genome sequencing have enabled real-time observation of mutation dynamics. Schreier et al. (41) developed a mutant fitness assay that identifies bacterial interactions in model ocean hot spots, demonstrating how ecological context shapes mutation selection in ways that laboratory monocultures cannot recapitulate.

### 8.2 Genome editing tools for bacterial mutation research

**Figure 4:**
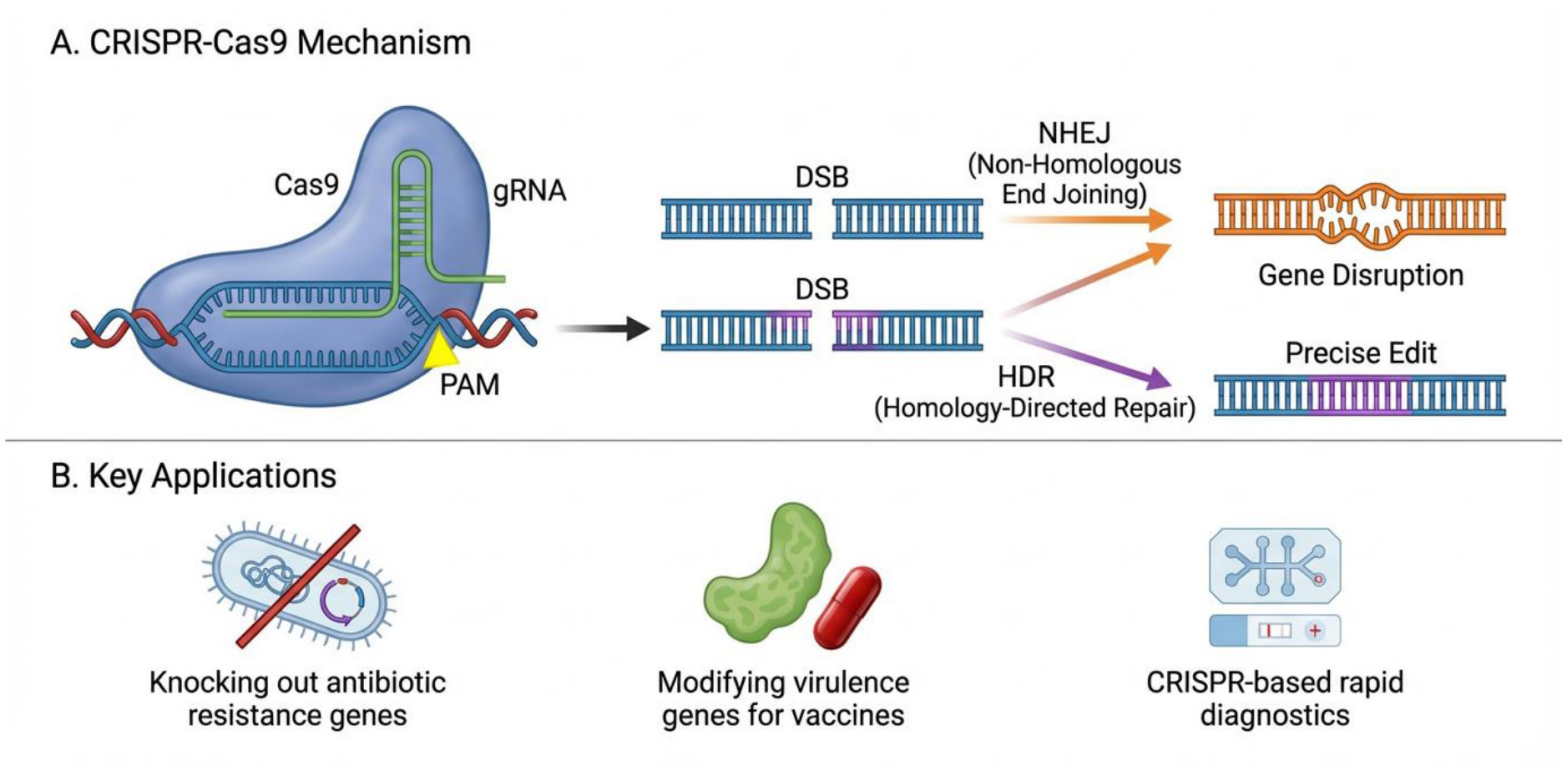
**(A)** CRISPR-Cas9 genome-editing mechanism showing Cas9-mediated DNA cleavage and subsequent repair through NHEJ or HDR pathways. **(B)** Key applications of CRISPR-Cas9 in bacterial research, including antibiotic resistance gene knockout, virulence gene modification for vaccine development, and rapid diagnostics.

There are many tools applied for genome editing in bacteria including:

**A: CRISPR-Cas9:** The primary goal of CRISPR-cas9 is to precisely target and eliminate antibiotic-resistant bacteria or to inactivate specific genes conferring resistance, thereby revitalizing conventional antibiotics and combating the spread of multi-drug-resistant infections. Scientists will be able to track the effects of CRISPR-Cas9 editing by eliminating particular DNA segments (Figure 4A). In bacterial research, CRISPR-Cas9 has been widely applied for the knockout of antibiotic resistance genes, modification of virulence-associated genes for vaccine development, and the establishment of rapid diagnostic platforms (Figure 4B). Additionally, a research investigation found that the target region indicated the mutation brought about by CRISPR-Cas9 breakage maintenance, which was not spontaneous (93). This finding allowed scientists to predict the mutational effect of CRISPRCas9 editing, allowing them to make precise modifications without the need for knock-ins (17, 94). Researchers can alter the *Mycobacterium tuberculosis* genome using genome-editing tools to look into genes associated with drug resistance or virulence. Even while there are still issues, including creating multiplexed CRISPR experiments to identify several mutations at once, developments are making the technology more useful for therapeutic applications. Particularly in areas where TB is still a major public health concern, integrating CRISPR into TB therapy could improve early identification, guide individualized therapy, and possibly aid in the development of more potent treatments (16). While CRISPRbased technologies offer powerful tools for investigating bacterial mutation mechanisms, several challenges remain. Off-target effects, limitations in delivery systems, and ethical considerations regarding the manipulation of microbial genomes must be carefully addressed before these technologies can be widely applied in clinical or environmental contexts. Furthermore, the evolutionary consequences of genome editing in microbial populations remain poorly understood and warrant further investigation (17).

**B: PCR-based Field Assays:** It is simple to convert current standard lab-based PCR tests into field-based monitoring assays thanks to the innovative integration of PCR with closed-tube and end-point detection of fluorescence. A variety of nucleic acid detection methods can be easily adapted and modified by incorporating specially developed 3D-printed elements into the procedure. These technologies directly enable real-time surveillance of genetic variation, including the emergence of new mutations in pathogens (95).

**C: Targeted Gene Disruption:** Numerous scientists are creating various strategies to create chromosomal gene disruption and deletions mutants, plasmid-bearing hybrid strains, and geneticsilencing techniques that allow the investigation of crucial genes (96).

### 8.3 Persister cells as reservoirs for mutation

It is important to study persister cells which are latent bacterial subpopulations that can withstand antibiotic treatment without developing genetic resistance by momentarily suspending metabolic activity. Their survival under antibiotic stress enables them to regenerate once the drug is removed, giving them time and opportunity to evolve new mutations. Over time, these alterations can become stable, changing temporary tolerance into genetic antibiotic resistance. Persister cells serve as a genetic reservoir, increasing the risk of future mutations through permitting survival and regrowth following antibiotic treatment, which allows resistance mutations to emerge and accelerates the emergence of drug-resistant strains (43). They don't possess their own resistance-causing genetic changes, but rather represent a latent community that can be chosen by antibiotics. Further research is required into understanding of persister cells which is critical for finding novel therapeutics to tackle persistent infections and prevent resistance from evolving. Persister cells have a distinct physiological state that distinguishes them from normal, constantly growing cells, stressing the need for innovative treatments that target these inactive cells directly (97).

## 9 Future perspectives and research gaps

Despite considerable advances in understanding bacterial mutation dynamics, several important research gaps remain. First, the environmental and host-associated factors that influence mutation rates in natural microbial communities remain incompletely characterized. Second, the relative contributions of mutation bias and natural selection in shaping bacterial evolutionary trajectories require further investigation using integrative experimental and computational approaches. Third, although emerging genomic technologies have significantly improved mutation detection, translating these findings into clinical strategies for predicting antimicrobial resistance remains challenging. Future research integrating evolutionary microbiology, genomics, bioinformatics, and clinical microbiology will be critical for developing predictive models of bacterial adaptation and for improving surveillance and early detection of antimicrobial resistance.

## 10 Conclusion

Mutations provide bacteria with new genetic variations that allow them to survive and adapt to rapidly changing environments. It is increasingly clear that when populations of pathogenic bacteria encounter environmental pressures, beneficial mutations with large effects on survival can arise, and mutator strains identified in clinical isolates may accelerate adaptive evolution. This review has critically synthesized current understanding of bacterial mutation dynamics, demonstrating that mutations are not merely random events but are shaped by predictable biases that influence evolutionary trajectories. To answer the central question posed in the Introduction: bacterial mutation dynamics—through spontaneous errors, induced changes, and bias—directly drive the evolution of virulence factors and antimicrobial resistance mechanisms. The key audience takeaway is that while mutations arise without regard to fitness (classical randomness), their non-uniform distribution (mutation bias) makes some adaptive outcomes more likely than others. This predictability opens avenues for anticipatory drug design and resistance management. It has been demonstrated that mutations promote the development of antibiotic resistance, permit phenotypic changes to evade detection and elimination by the immune response of the host, and permit the transition to cellular forms that are more viable in the particular setting. The clinical relevance of these findings is increasingly evident, with recent studies documenting resistance evolution during patient treatment and revealing unexpected cross-resistance patterns that complicate therapeutic decisions. These observations highlight the urgent need for a deeper understanding of mutation-driven evolutionary processes in pathogenic bacteria and their implications for clinical treatment strategies.

The majority of mutators that have been identified so far have mutations in defective repair pathway elements. It is evident, therefore, that other genetic mechanisms in prokaryotes may also contribute to the emergence of mutator phenotypes, but these have not yet been fully identified. Recent research has started to clarify the genetic variables controlling the phenotypic changes seen in mutators and emphasizes a crucial adaptation concept: during the initial phases of adaptation, mutations frequently take place in global regulatory mechanisms or signaling pathways that have multiple consequences. Emerging technologies including CRISPR-based editing, machine learning prediction, and single-cell sequencing are revolutionizing our ability to study and potentially direct mutation outcomes, offering new hope for combating antimicrobial resistance. These advanced analytical approaches provide unprecedented opportunities to investigate mutation dynamics at the genomic and single-cell levels, enabling researchers to better predict evolutionary trajectories and identify potential therapeutic targets. Our knowledge of pathogenicity and ecological niche adaptability will be substantially improved by additional research exploring these genotypic changes. Ultimately, integrating evolutionary microbiology, genomics, and clinical research will be essential for translating advances in mutation biology into effective strategies to combat antimicrobial resistance and improve global infectious disease management.
